# List of Reviewers

**DOI:** 10.1017/jns.2023.112

**Published:** 2024-01-09

The Editorial Board of the Journal of Nutritional Science would like to thank the following for their contribution as peer reviewers in 2023:

- Abate, Bedilu Abebe
- Abbas, Mateen
- Abu Jadayil, Seham
- Abu-Zaid, Ahmed
- Adebayo, Folasade
- Akter, Fahmida
- Almeida, Cláudia
- Amha, Haile
- Amoah, Isaac
- Anbu Ananthan, Velmurugan
- Andersen, Stig
- Astutik, Erni
- Attia, Suzanna
- Ayusari, Amelya
- Babel, Riddhi
- Bailey, Christopher
- Bastihalli Tukaramrao, Diwakar
- Batistia, Sueny
- Bekele, Tesfaye
- Bener, Abdulbari
- Bennett, Annemarie
- Berhane, Anteneh
- Bermudez-Millan, Angela
- Bernardes, Simone
- Bird, Julia
- Bjerregaard, Anne
- Borlu, Arda
- Bostrom, Anne-Marie
- Bountziouka, Vasiliki
- Brown, Teresa
- Buffini, Maria
- Calder, Philip C.
- Caldwell, Julia
- Canas, Jose
- Cao, Dedong
- Carvalho, Januse
- Casperson, Shanon
- Chan, Kara
- Chan, Ronna
- Choukri, Maria
- Cobucci, Ricardo
- Crowley, Jennifer
- Cuadrado-Soto, Esther
- Da Costa, Teresa Helena
- Daniel, A.
- Davies, Ian
- Denli, Muzaffer
- Diddana, Tona
- Dolatkhah, Neda
- Doledec, David
- Dominguez Perez, Ruben Abraham
- Elsahoryi, Nour
- Elshahat, Sarah
- Emmett, Pauline
- Endale, Fisum
- Engidaw, Melaku
- English, Marcia
- Faber, Mieke
- Feleke, Fentaw
- Fisera, Miroslav
- Florez, Karen R.
- Freitas, Ana Tereza
- Fujita, Hiroyuki
- Fujiwara, Aya
- Gasier, Heath
- Gildestad, Trude
- Girma, Helen
- Gonçalves, Danielle
- Gonzales, Gerard Bryan
- Gosdin, Lucas
- Gutierrez-Silerio, Gloria Yareli
- Hammouh, Fadwa
- Haselow, Nancy Jean
- Hasman, Andreas
- Hekmatdoost, Azita
- Henrina, Joshua
- Heshmati, Javad
- Hess, Julie M.
- Ho, Yuh-Shan
- Hollis-Hansen, Kelseanna
- Hosseinzadeh-Attar, Mohammad Javad
- Hruby, Adela
- Imohe, Annette
- Itkonen, Suvi
- Iwaniec, Urszula T.
- James, Philip
- Jima, Beshada
- Jitrapakdee, Sarawut
- Jugha, Vanessa
- Juul, Filippa
- Karupaiah, Tilakavati
- Kearney, John
- Kebede, Tsehay
- Khamis, Ahmed
- Kokol, Peter
- Kontogianni, Meropi
- Kontor, Enikő
- Kord-Varkaneh, Hamed
- Krupa-Kozak, Urszula
- Larson-Meyer, D. Enette
- Lin, Sophia
- Loperfido, Federica
- Louca, Bano
- Lovell, Amy
- Maeda, Hayato
- Martin-del-Campo, Fabiola
- Mascherini, Gabriele
- Matos, Juliana
- Mbhenyane, Xikombiso
- Medin, Anine
- Mengie, Muluye Gebrie
- Mezgebu, Getachew
- Micic, Ruzica
- Mistura, Lorenza
- Mogre, Victor
- Mohammadnezhad, Masoud
- Myszkowska-Ryciak, Joanna
- Nakamura, Mieko
- Nikbakht Jam, Irandokht
- Niseteo, Tena
- O'Donovan, Sarah
- Okuda, Nagako
- Orces, Carlos
- O'Reilly, Eilis
- Paknahad, Zamzam
- Palchetti, Cecilia
- Pan, Dorothy
- Parker, Christina
- Pentieva, Kristina
- Petros, Legese
- Plichta, Marta
- Rehkopf, David H.
- Reicks, Marla
- Renteria-Mexia, Ana
- Rizzo, Gianluca
- Rogers, Imogen
- Rosendahl-Riise, Hanne
- Saaka, Mahama
- Saravia, Luisa
- Scoditti, Egeria
- Sidnell, Anne
- Silva, Débora
- Singh, Jitendra
- Solovyev, Nikolay
- Stefan, Norbert
- Steyn, Nelia
- Stotz, Sarah
- Sugimoto, Koki
- Tomczak, Andrzej
- Tuccinardi, Dario
- Tümer, Güzin
- Tups, Alexander
- Ulusoy Gezer, Hande
- Vaiman, Daniel
- Van den Heuvel, Emmy
- Wang, Kimberley C. W.
- Wang, Zeneng
- Weschenfelder, Camila
- Wieringa, Frank
- Windus, Janelle
- Yahia, Najat
- Yan, Alice F.
- Yan, Jin
- Yaxley, Alison
- Zhang, Tianyi
- Zhang, Yong
- Zheng, Xiaoyun
- Zhou, Xihong

